# Tebuconazole Toxicity in the Parotid Gland of Adolescent Rats

**DOI:** 10.1002/jbt.70833

**Published:** 2026-04-10

**Authors:** Gabriel Carvalhal de Aguiar, Lorrany da Silva Avanci, Daniel Vitor de Souza, Lucas Vilas‐Bôas Correia, Regina Cláudia Barbosa da Silva, Flavia de Oliveira, Andrea Cristina de Moraes Malinverni, Fernando Augusto Cintra Magalhães, Daniel Araki Ribeiro

**Affiliations:** ^1^ Department of Biosciences, Institute of Health and Society, UNIFESP Federal University of São Paulo São Paulo Brazil; ^2^ Department of Pathology, Paulista Medical School, UNIFESP Federal University of São Paulo São Paulo Brazil

**Keywords:** apoptosis, inflammation, mouse, oxidative stress, parotid, proliferation, salivary glands, tebuconazole, tissue remodeling

## Abstract

This study aims to investigate the effects of subchronic exposure to Tebuconazole (TEB) on the parotid salivary gland of adolescent rats. These animals were exposed daily to TEB (10, 20, and 50 mg/kg, orally) or vehicle for 30 days. The parotid glands were evaluated by histopathological (hematoxylin‐eosin staining and histomorphometry), histochemical (Periodic Acid‐Schiff [PAS] for glycoconjugates and Picrosirius Red for collagen fibers) and immunohistochemical analysis for markers of oxidative stress (8‐OHdG), inflammation (COX‐2), apoptosis (caspase‐3), epithelial remodeling (CK7), and cell proliferation (Ki‐67). Histopathological results showed dose‐dependent morphological changes, including architectural disorganization of the glandular parenchyma, cytoplasmic vacuolation, and inflammatory infiltrate. PAS results showed a significant reduction in labeling in the 50‐ and 20‐mg/kg groups in relation to the 10 mg/kg and control groups, evincing a reduction in cell activity. The immunostaining of 8‐OHdG (*p* < 0.05 at the 50 mg/kg dose), caspase‐3 (*p* < 0.05 at all doses), CK7 (*p* < 0.05 at the 20‐ and 50‐mg/kg doses), and COX‐2 (*p* < 0.05 at all doses) significantly increased. The expression of the proliferation marker Ki‐67 showed no significant alteration, whereas the histomorphometric analysis confirmed a progressive and significant reduction in cell density (*p* < 0.05). Moreover, the Picrosirius Red staining technique showed an increase in fibrotic tissue within the glandular parenchyma. In conclusion, this study found that the subchronic exposure to TEB induces dose‐dependent toxicity in the parotid gland of adolescent rats.

## Graphical abstract

Exposure to tebuconazole induces dose‐dependent toxicity in the parotid gland of Wistar rats, through genomic oxidative stress, inflammatory processes, and apoptosis.

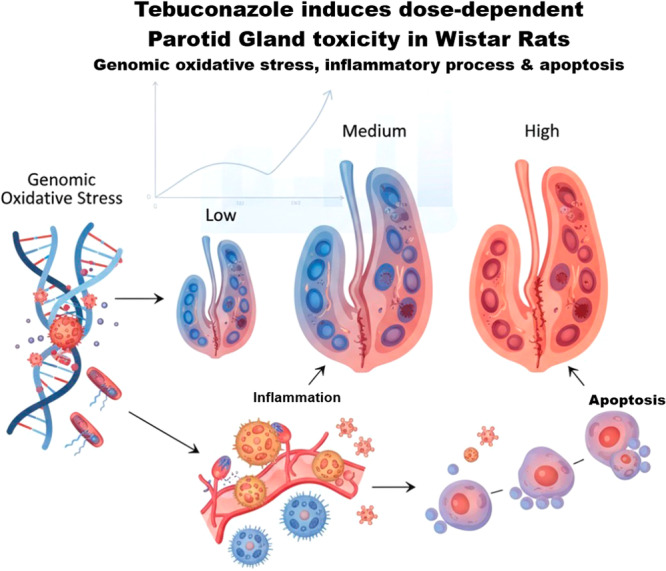

## 1 Introduction

The triazole tebuconazole [1‐(4‐chlorophenyl)‐4,4‐dimethyl‐3‐(1,2,4‐triazole‐1‐ylmethyl)‐pentan‐3‐ol] (TEB) is, globally, one of the most used systemic fungicides in agriculture to control fungal diseases in crops composing the human diet, such as cereals, fruits, and vegetables [1]. Its effectiveness lies in its ability to inhibit the fungal enzyme sterol‐14‐demethylase, which is crucial for the synthesis of ergosterol, a vital component of the fungal cell membrane [2]. Despite its predominant agricultural use, it also serves as a biocide to conserve materials as wood [3].

The wide and continuous use of TEB results in the significant presence of its residues in the environment, contaminating the soil and water, and, consequently, the food, representing a potential risk to human health [4]. The main route of human exposure is dietary, via the consumption of contaminated food, but occupational exposure also poses a significant risk. For instance, one case report described a farmer who developed acute toxic hepatitis after handling TEB without personal protective equipment [5]. A report from the Brazilian National Health Surveillance Agency showed that, between 2017 and 2018, 570 of the 4616 analyzed food samples (12% of the total) showed TEB residues, including popular items such as rice, beans, wheat, and oranges [6].

Agencies worldwide have concerned themselves with TEB since the Environmental Protection Agency classified it as a carcinogen in 1993. In 2024, Health Canada reduced TEB application rates due to unacceptable exposure risks [7]. Confirmation of human exposure stems from the detection of TEB in farmers’ urine and hair samples, indicating its systemic effect [8]

Once absorbed the liver bio transforms TEB and distributes it to various organs, acting as a multisystem xenobiotic [9]. The scientific literature has shown its adverse effects on various tissues. The liver configures a primary target, and studies have shown that TEB induces oxidative stress and lipid accumulation in liver cells [10], a risk confirmed by case reports of acute hepatotoxicity in humans occupationally exposed to it [5]. Previous studies also point to its toxic action on the heart as it can electromechanically and structurally change the organ in rats [11, 12], and cause oxidative damage and apoptosis in kidney cells [13].

Despite the extensive knowledge about its systemic toxicity, the impact of TEB on exocrine glands, such as salivary glands, remains underexplored. Exposure during critical developmental windows, such as adolescence, is of particular concern, as food consumption per kilogram of body weight is significantly higher in this period than in adults, increasing exposure. The documented negative impacts, the risk to human health due to the consumption of TEB residues, and the absence of studies on degenerative changes in salivary glands associated with exposure to this contaminant evince the relevance of new studies regarding this triazole.

## 2 Materials and Methods

### 2.1 Reagents

The TEB‐based compound (CAS Number: 107534‐96‐3, purity = 43%, Zafiro), which was obtained from the company Diagro S.A., was diluted in corn oil to make the solutions for the treatments. The doses of the contaminant were adjusted to the final volume of 1 mL/kg of body weight of the animals and were chosen based on Yang et al. [14].

### 2.2 Animals

A total of 32 3‐week‐old male Wistar rats, from the Center for the Development of Experimental Models for Biology and Medicine of the Federal University of São Paulo (CEDEME/Unifesp), weighing from 50 to 55 g at the beginning of the experiment, were used. The choice of adolescent rats as an experimental model in this study was a strategic decision to evaluate the effects of TEB during a developmental window. The animals were allocated in groups of four individuals per cage with water and food ad libitum. They were kept in a vivarium under artificial lighting, with a 12‐h light–dark cycle starting at 7 a.m., and constant temperature (23 ± 1°C). After 7 days of habituation to the laboratory conditions, the rats were subjected to a protocol of exposure to TEB. All experiments were carried out in the light cycle of the day.

### 2.3 Treatments

The administration of TEB was started on the 28th postnatal day up to the 57th, a period equivalent to adolescence in rats, according to Schneider [15].

In this study, four experimental groups were established, each composed of eight animals (Table 1). Each animal was exposed to a single concentration of TEB.

**Table 1:** Assignment of experimental groups and their respective concentrations of TEB.

Experimental groups	TEB concentrations
**Group 1**	Corn oil (TEB control vehicle group)
**Group 2**	Corn oil + 10 mg/kg TEB (low dose)
**Group 3**	Corn oil + 20 mg/kg TEB (medium dose)
**Group 4**	Corn oil + 50 mg/kg TEB (high dose)

### 2.4 Oral Administration

The rats were exposed daily, for 30 consecutive days, to the vehicle solution or TEB at concentrations of 10, 25, or 50 mg/kg per gavage [16]. Moreover, before inserting the tip of the gavage cannula into the oral cavity of the rodents, it was soaked with a 10% sucrose solution, as it contributes to reducing stress and improving the well‐being of the animals during the procedure [16].

### 2.5 Histopathological Analysis

The parotid glands were fixed in 10% buffered formalin for 24 h, gradually dehydrated in alcohol, diaphanized in xylol, and later incorporated into paraffin blocks. The tissues were cut into 4‐μm thick sections, dewaxed in xylol, rehydrated in ethanol (99.5%), and stained with hematoxylin‐eosin (HE) for the histopathological evaluation.

The tissues were analyzed according to the presence of inflammatory infiltrates, vascular congestion, pseudocysts, tissue disorganization, hemorrhage, signs of apoptosis, areas of necrosis, hyperplasia, metaplasia and neoplastic lesions, following a semi‐quantitative method that adopted the criteria of severity, intensity, and extent of histopathological changes, which were scored as: (0) preservation of tissue integrity; (1) vacuolar degeneration and inflammatory infiltrate; (2) tissue disorganization and hemorrhage; and (3) death cells and pseudocysts.

From the hematoxylin‐eosin (HE) stained slides used in the histopathological analysis, the histomorphometric evaluation was performed to quantify the cell density of the glandular tissue (cells/1000 μm²) [17].

### 2.6 Immunohistochemistry

To perform this technique, the protocol in Moretti et al. [18] was used to evaluate markers of cell proliferation (Ki‐67) (Biocare Medical, USA—1:150), apoptosis (cleaved caspase‐3), oxidative stress (8‐hydroxy‐2′‐deoxyguanosine—8‐OHdG), epithelial remodeling (cytokeratin 7—CK7) (Santa Cruz Biotechnology, USA—1:500), and inflammation (cyclooxygenase‐2—COX‐2) (Abcam—1:100). The 3‐μm histological sections were deparaffinized in xylol, rehydrated in ethanol (99.5%), and pretreated with a citric acid buffer solution (10 nM, pH 6, 0.1 M citric acid—*Synth*; 0.1 M sodium citrate—*Synth*, São Paulo, Brazil) in microwaves for three 5‐min cycle, for antigenic recovery. Then, the sections were incubated with 3% hydrogen peroxide (H_2_O_2_) to inactivate endogenous peroxidases. Nonspecific proteins were then blocked with Protein Blocker (*Starr Trek Universal HRP Detection, Biocare Medical*) for 30 min to avoid possible cross‐links. Then, the histological sections were washed in distilled water and incubated with the primary antibodies. The antigen‐antibody complexes formed were visualized with a 0.05% DAB (3,3‐diaminobenzidine) chromogen solution (*DAKO North America, Inc, California, USA*) and stained with hematoxylin (8‐OHdG, caspase‐3, COX, and CK7) or Fast‐Green (Ki‐67).

The immunohistochemical evaluation was performed according to the antibody. Anti‐Ki‐6 expression was analyzed by a semi‐quantitative method in which the frequency of immunopositive cells were counted in a 10‐field count. The protein expression of anti‐8‐OHdG, cleaved anti‐caspase‐3, anti‐COX2, and anti‐CK7 was evaluated by a semiquantitative method, scoring marking extension thus: (0) no marking, (1) <10% marking extension, (2) 10%–50% marking extension, and (3) >50% marking extension.

### 2.7 Periodic Acid‐Schiff (PAS)

After histological processing, the histological sections of the salivary glands were deparaffinized, hydrated, and oxidized in a periodic acid solution at 0.5% for 15 min. After washing in distilled water, the slides were incubated with Schiff's reagent for 30 min in an environment protected from light and then washed in distilled water. Counterstaining was done with Fast Green for 5 s to provide contrast to the PAS‐negative tissue components. Finally, the slides were dehydrated in an increasing series of ethanols, diaphanized in xylol, and assembled.

The neutral glycoconjugates (glycogen) were semiquantitatively evaluated, scoring the criteria for the extension of PAS‐positive labeling classified as: (0) <10% of labeling extension, (1) 11%–30% marking length, (2) 31%–60% marking length, and (3) >61% markup length.

### 2.8 Collagen Birefringence Analysis

To analyze the density and organization of collagen fibers, 4‐μm thick histological sections from parotid glands that were embedded in paraffin were deparaffinized in xylol and rehydrated in a decreasing series of ethanol. Then, the slides were immersed in the Picrosirius Red solution for 20 min for the specific staining of collagen fibers. After staining, the sections were washed in distilled water and counterstained with Harris’ hematoxylin for 5 min to highlight the nuclei of the acinar and ductal cells. Finally, the slides were dehydrated in an increasing series of ethanol, diaphanized in xylol, and mounted with coverslips using Entellan resin.

The collagen fibers were qualitatively and comparatively evaluated under an optical microscope with polarized light filters considering the accumulation and thickening of connective tissue, especially on the interlobar, perivascular, and periductal septum regions between the control and treated groups, in addition to the characterization and predominance of collagen fiber types based on their birefringence. Type I collagen fibers, which are thicker and associated with fibrosis, showed yellow, orange, or red birefringence colors, whereas type III collagen fibers, which are thinner and associated with early phases of healing and repair, showed green coloration.

### 2.9 Statistical Analysis

All results are shown as mean ± standard deviation. To obtain the results from the semiquantitative and quantitative analyses, the ANOVA test, followed by the Tukey's post hoc test were used in parametrically distributed data and the Kruskal‐Wallis test, followed by the Dunn's multiple comparison test were used for non‐parametric data. Statistical calculations were performed on Jamovi 2.4.5. A significance level of 95% (*p* < 0.05) was adopted.

## 3 Results

### 3.1 Histopathological Analysis

The histopathological evaluation showed significant dose‐dependent morphological changes in the groups exposed to TEB (Table 2). Nuclear alterations such as apoptosis only occurred in the treated groups, with similar frequency between doses. Also, tissue architectural disorganization significantly increased from the 10 mg/kg dose upward.

**Table 2:** Total scores of histopathological alterations of the parotid salivary gland of Wistar rats exposed to tebuconazole.

Groups (*n* = 8)	*p* < 0.05	Score 0	Score 1	Score 2	Score 3
**Control**	—	8	0	0	0
**10‐mg/kg**	(*)	0	3	4	1
**20‐mg/kg**	(*)	0	3	0	2
**50‐mg/kg**	(*)(**)	0	0	1	7

*Note:* (*)*p *< 0.05 when compared to the control group. (**)*p *< 0.05 when compared to the 10‐mg/kg group.

**Figure 1:**
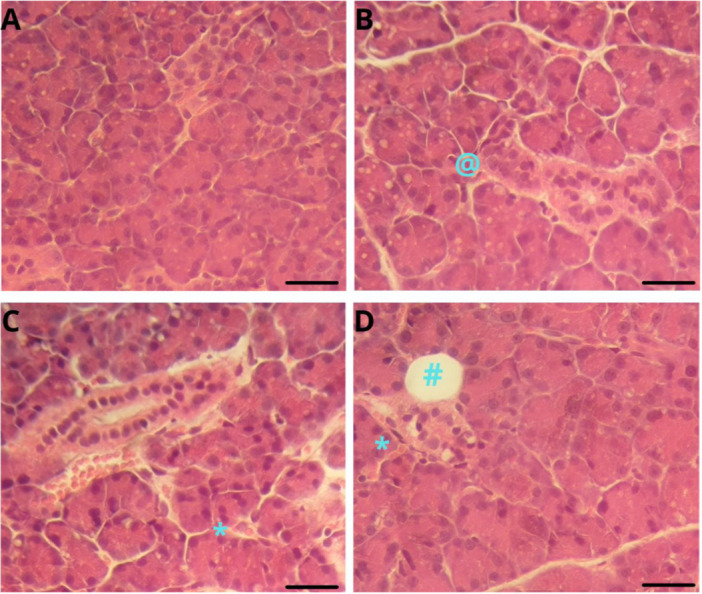
Photomicrograph of the parotid salivary gland stained in HE. (A) represents the glandular tissue of the control group, with no morphological changes. (B) represents the glandular tissue of the 10‐mg/kg group, with vacuolation. (C) represents the glandular tissue of the 20‐mg/kg group, with intercellular erythrocytes. (D) represents the glandular tissue of the 50‐mg/kg group, with pseudocyst and intercellular erythrocytes. The alterations were evidenced in (@) vacuolation, (*) hemorrhage, and (#) pseudocysts. Scale bar = 50 μm.

All treated groups showed significantly higher hemorrhage than the control group, tending toward greater severity at the 50‐mg/kg dose, suggesting impairment of vascular integrity. Similarly, the formation of intracytoplasmic vacuoles showed a significant increase in all exposed groups, characterizing hydropic degeneration. Higher doses caused more pronounced inflammatory infiltrates, showing an inflammatory response proportional to the intensity of the tissue injury. Only the treated animals showed pseudocysts, with greater frequency in the group subjected to 50 mg/kg a day, possibly reflecting areas of tissue reorganization. These findings suggest that exposure to TEB promotes relevant dose‐dependent histopathological changes that match progressive degenerative, inflammatory, and structural processes (Figure 1).

### 3.2 Histomorphometry Analysis

**Figure 2:**
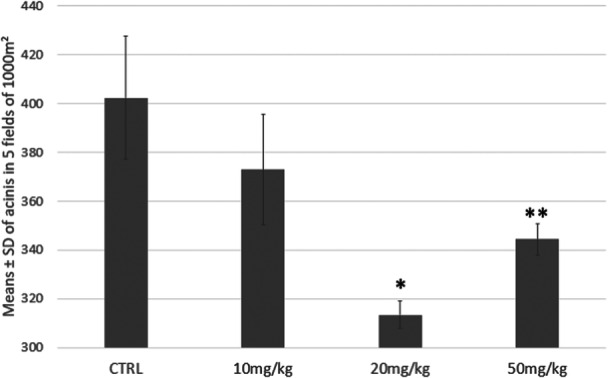
Mean ± PD of acini in five fields of 10,000 μm² of parotid salivary glands in Wistar rats exposed to tebuconazole (*n* = 8). Significant changes with *p* < 0.05 compared to the control group and 10 mg/kg (*) and compared to the control group, 10‐mg/kg and 20‐mg/kg (**).

The histomorphometric analysis of the parotid salivary gland showed a progressive and statistically significant reduction in cell density (cells/10,000 μm²) in the treated groups when compared with control (Figure 2). The10‐mg/kg group showed no significant differences. The 20‐mg/kg group showed a drastic and statistically different decrease in relation to control (*p* < 0.05) and the 10 mg/kg group (*p* < 0.05). In the group under the highest dose (50 mg/kg), the decrease remained significant in comparison with the control and 10 mg/kg groups, showing a significant increase when compared to the 20‐mg/kg group (*p* < 0.05). These findings indicate that exposure to TEB promotes histomorphometric changes that indicate cell loss or structural reorganization of glandular tissue.

### 3.3 Immunohistochemical Analysis

#### 3.3.1 Ki‐67

**Figure 3:**
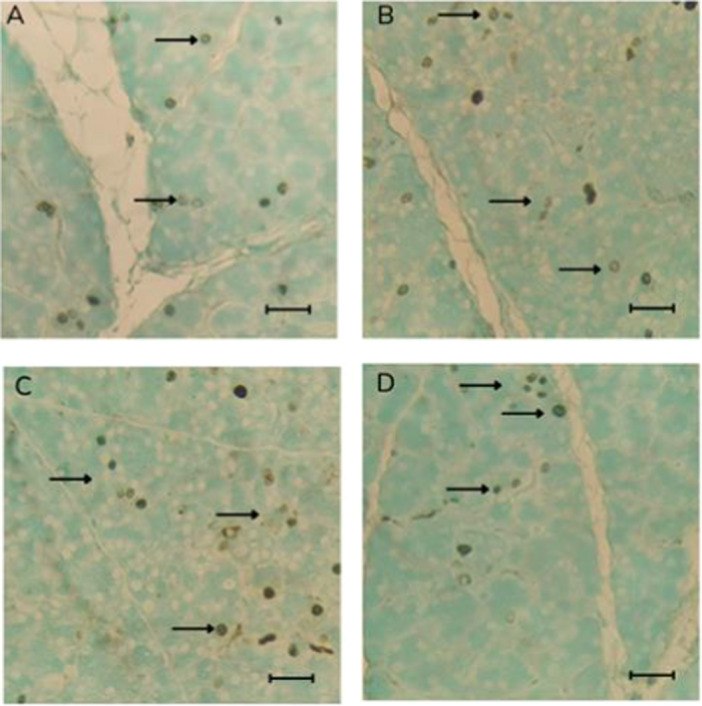
Photomicrograph of Ki‐67 immunostaining in parotid salivary glands of Wistar rats exposed to tebuconazole, in the following groups: (A) Control, (B) 10 mg/kg group, (C) 20 mg/kg group, and (D) 50‐mg/kg. The arrows indicate marked cores. Scale bar = 50 μm.

**Figure 4:**
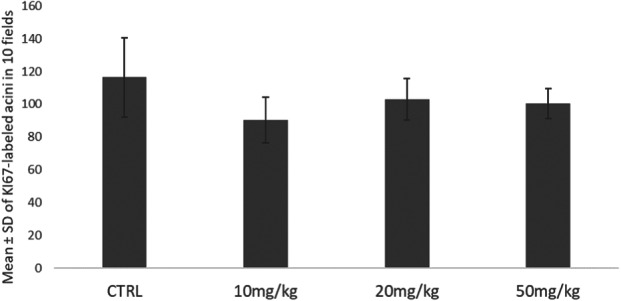
Mean ± SD of the total number of Ki‐67 labeled nuclei in 10 fields in parotid salivary glands in Wistar rats exposed to tebuconazole (*n* = 5).

The immunohistochemical analysis for Ki‐67, a marker of cell proliferation, of parotid salivary gland samples showed no statistically significant differences between the experimental groups (Figure 4). The control and the 10, 20, and 50 mg/kg groups showed similar mean numbers of nuclei labeled in five fields (Figure 3), indicating that exposure to the fungicide failed to significantly change the proliferative activity of glandular cells.

#### 3.3.2 8‐OHdG

**Figure 5:**
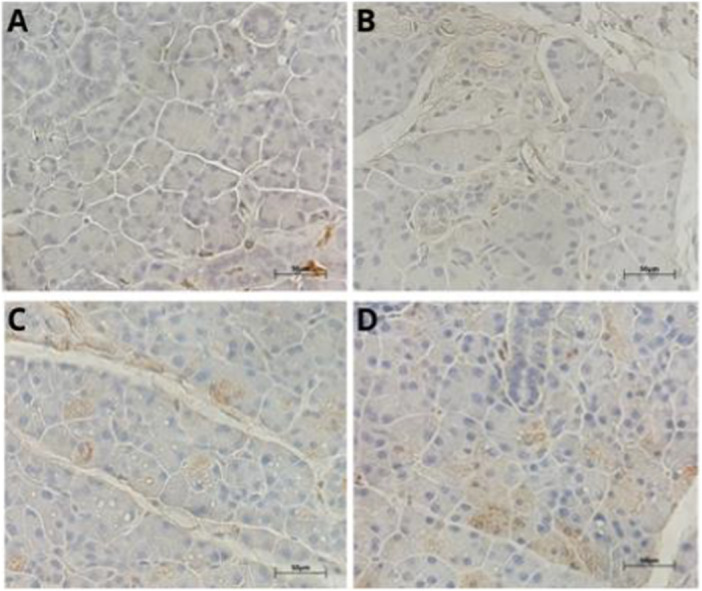
Photomicrograph of 8‐OHdG immunostaining in the parotid salivary glands of Wistar rats exposed to tebuconazole, in groups (A) Control, (B) 10‐mg/kg, (C) 20‐mg/kg, and (D) 50‐mg/kg. Scale bar = 50 μm. *Note:* Cytoplasm of acini marked in brown.

The immunohistochemical analysis for 8‐OHdG, a marker of oxidative DNA damage, showed a progressive increase in immunostaining according to the increase in doses (Figure 5). The animals in the control group showed low marking, whereas those in the exposed groups gradually shifted to intermediate scores.

Only the 50 mg/kg group showed a significant increase in the number of animals with higher scores, with a significant difference (*p *< 0.05) in relation to the control (Table 3). These findings indicate that exposure increased oxidative stress, as per the greater formation of DNA oxidation products, especially at higher doses.

**Table 3:** 8‐OHdG immunostaining scores per animal in the parotid gland of Wistar rats exposed to tebuconazole.

Groups (*n* = 5)	*p* < 0.05	Score 0	Score 1	Score 2	Score 3
**Control**	—	3	2	0	0
**10‐mg/kg**	—	2	3	0	0
**20‐mg/kg**	—	1	4	0	0
**50‐mg/kg**	(*)	0	2	3	0

*Note:* (*)*p *< 0.05 when compared to the control group.

#### 3.3.3 COX‐2

**Figure 6:**
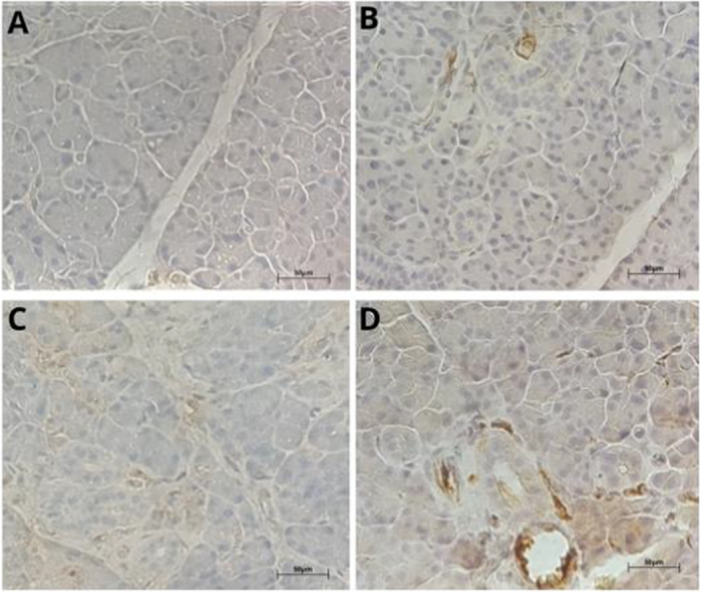
Photomicrograph of COX‐2 immunostaining in the parotid salivary glands of Wistar rats exposed to tebuconazole, in the groups: (A) Control, (B) 10‐mg/kg group, (C) 20‐mg/kg group, and (D) 50‐mg/kg. The arrows indicate marked cores. Scale bar = 50 μm.

Immunostaining for cyclooxygenase‐2 (COX‐2), an enzyme associated with inflammatory processes, showed a significant increase in its expression in the treated groups. The absence of labeling predominated in the control, whereas all treated groups showed a redistribution of scores to intermediate patterns (Figure 6), indicating an increase in local inflammatory responses.

This statistically significant increase (*p *< 0.05) at all doses suggests that activation of the inflammatory pathway occurs even under less intense exposure (Table 4). These findings indicate that exposure to TEB induces an early and sustained inflammatory response in the parotid gland.

**Table 4:** COX‐2 immunostaining scores per animal in the parotid gland of Wistar rats exposed to tebuconazole.

Groups (*n* = 5)	*p* < 0.05	Score 0	Score 1	Score 2	Score 3
**Control**	—	3	2	0	0
**10‐mg/kg**	(*)	0	3	2	0
**20‐mg/kg**	(*)	0	2	3	0
**50‐mg/kg**	(*)	0	3	2	0

*Note:* (*)*p *< 0.05 when compared to the control group.

#### 3.3.4 Cleaved Caspase‐3

**Figure 7:**
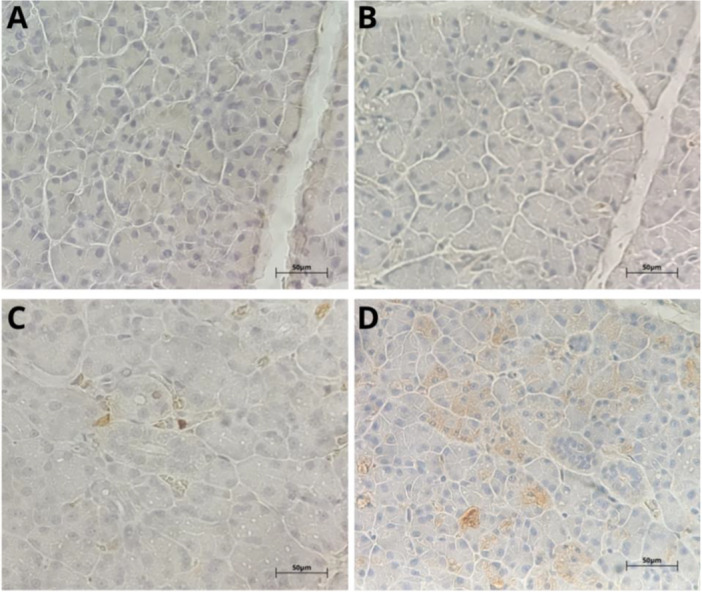
Photomicrograph of cleaved Caspase‐3 immunostaining in the parotid salivary glands of Wistar rats exposed to tebuconazole, in groups (A) Control, (B) 10‐mg/kg, (C) 20‐mg/kg, and (D) 50‐mg/kg. Scale bar = 50 μm.

Immunostaining for cleaved caspase‐3, a marker associated with the apoptosis pathway, showed a significant increased expression in the TEB‐treated groups. The absence of marking predominated in the control, and all treated groups showed redistribution to intermediate scores, evincing increased apoptotic activity (Figure 7).

This increase was statistically significant (*p *< 0.05) in all treated groups, suggesting that even low‐intensity exposure activates the apoptotic pathway (Table 5). These findings indicate that the substance induces apoptosis consistently and early, a response that persists at higher doses.

**Table 5:** Caspase‐3 immunostaining scores cleaved by animal in the parotid gland of Wistar rats exposed to tebuconazole.

Groups (*n* = 5)	*p* < 0.05	Score 0	Score 1	Score 2	Score 3
**Control**	—	4	1	0	0
**10‐mg/kg**	(*)	0	2	3	0
**20‐mg/kg**	(*)	0	3	2	0
**50‐mg/kg**	(*)	0	3	2	0

*Note:* (*)*p* < 0.05 when compared to the control group.

#### 3.3.5 CK7

The immunohistochemical analysis showed a significant increase in CK7 expression in the groups under the highest doses of TEB. Low immunostaining predominated in the control group, whereas in the treated groups, especially those above 20 mg/kg, a shift to intermediate and high levels scores occurred (Table 6).

**Table 6:** CK7 immunostaining scores per animal in the parotid gland of Wistar rats exposed to tebuconazole.

Groups (*n* = 5)	*p* < 0.05	Score 0	Score 1	Score 2	Score 3
**Control**	—	2	2	0	0
**10‐mg/kg**	—	0	4	0	0
**20‐mg/kg**	(*)	0	1	2	1
**50‐mg/kg**	(*)	0	0	3	1

*Note:* (*)*p *< 0.05 when compared to the control group.

**Figure 8:**
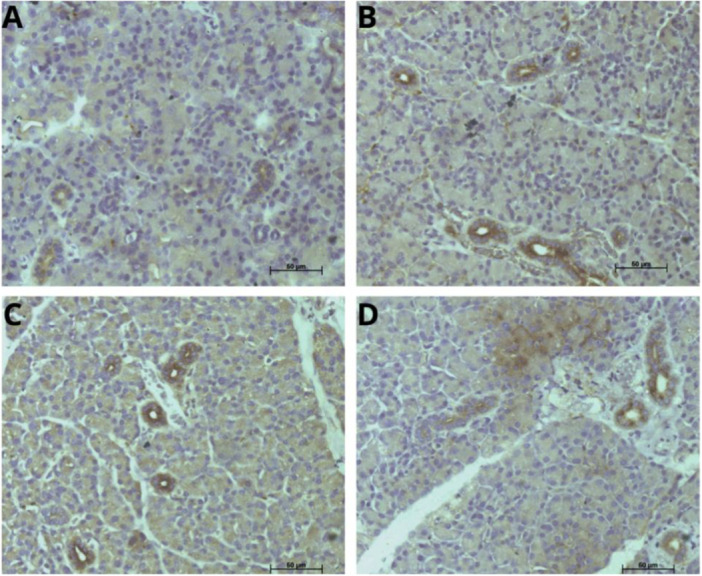
Photomicrograph of CK7 immunostaining in the parotid salivary glands of Wistar rats exposed to tebuconazole, in groups (A) Control, (B) 10‐mg/kg, (C) 20‐mg/kg, and (D) 50‐mg/kg. Scale bar = 50 μm.

The 20‐ and 50‐mg/kg groups showed statistically significant differences (*p *< 0.05) when compared with the control, showing that exposure changed the expression of the intermediate filaments associated with the glandular epithelium (Figure 8). This pattern may be related to processes of cellular adaptation in the face of tissue damage and alterations in cell differentiation due to chemical damage.

### 3.4 Histochemistry (Periodic Schiff Acid)

Histochemical analysis with PAS staining showed a significant reduction in the content of PAS‐positive substances, such as glycogen and glycoproteins, in the groups under the highest doses of TEB. The control showed a more accentuated marking, whereas scores of the treated ones, especially from 20 mg/kg onward, shifted to lower levels, indicating a loss of PAS‐positive material (Table 7).

**Table 7:** PAS‐positive acini staining scores per animal in the parotid gland of Wistar rats exposed to tebuconazole.

Groups (*n* = 5)	*p* < 0.05	Score 0	Score 1	Score 2	Score 3
**Control**	—	0	0	2	3
**10‐mg/kg**	—	0	2	3	0
**20‐mg/kg**	(*)	1	4	0	0
**50‐mg/kg**	(*)(**)	3	2	0	0

*Note:* (*)*p* < 0.05 when compared to the control group. (**)*p* < 0.05 when compared to the 10 mg/kg group.

**Figure 9:**
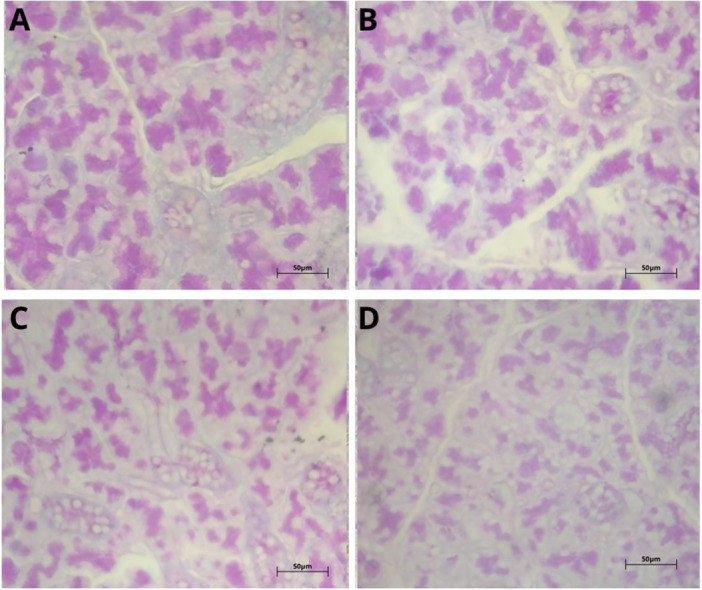
Photomicrograph of the marking of PAS‐positive acini in the parotid salivary glands of Wistar rats exposed to tebuconazole, in groups (A) Control, (B) 10‐mg/kg, (C) 20‐mg/kg, and (D) 50‐mg/kg. Scale bar = 50μm.

The 20‐ and 50‐mg/kg groups showed statistically significant differences (*p *< 0.05) when compared to the control. Notably, the 50 mg/kg group also showed a significant reduction in relation to the 10 mg/kg group, showing that exposure to the substance depleted carbohydrate stores in the glandular tissue (Figure 9). This pattern is consistent with a cellular stress response and may indicate an altered energy metabolism or secretory function of cells in response to chemical damage.

### 3.5 Collagen Birefringence Analysis

Analysis of the parotid glands stained with Picrosirius Red under polarized light showed an accumulation of connective tissue in the groups exposed to TEB when compared with the control. This accumulation mainly occurred in the interlobular septa and around vessels and secretory ducts.

**Figure 10:**
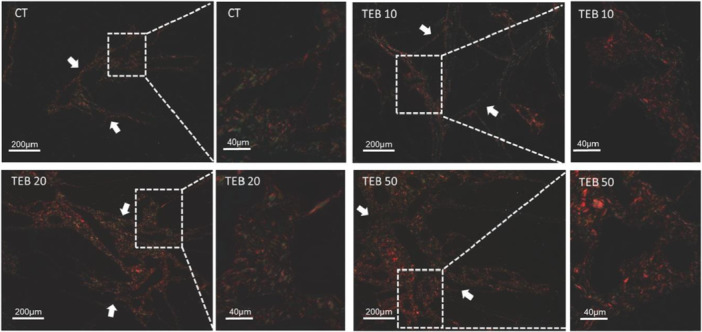
Photomicrograph under polarized light of the marking of type I and type III collagen fibers of parotid salivary gland slides stained by the Picrosirius Red technique. (A and A.1) Control group, (B and B.1) 10‐mg/kg TEB group, (C and C.1) 20‐mg/kg TEB group, (D and D.1) 50‐mg/kg. (A, B, C, D) 40× magnification. Scale bar = 200 μm; (A.1, B.1, C.1, and D.1) Scale bar = 40 μm. White arrows: indicate thickening of interlobular connective tissue.

Perivascular and periductal connective tissue thickening (indicating fibrosis) showed a dose‐dependent relation, being more pronounced in the groups that received higher doses of TEB (10, 20, and 50 mg/kg) when compared to the group. The analysis of the connective tissue mesh found type I (stained in yellow, orange, and red) and type III collagen fibers (stained in green). Notably, the 50‐mg/kg group showed was a predominance of type I collagen fibers, which are characteristically thicker and prevalent in fibrotic tissues (Figure 10).

## 4 Discussion

The extensive use of TEB (a common fungicide in agriculture) raises concerns about its possible toxic effects on non‐target organisms. While its toxic impacts on organs such as the liver, kidneys, and heart are relatively well known, we have little understanding of how this compound affects glandular tissues, particularly salivary glands. The results of this study offer a relevant and unprecedented contribution to this context by exploring the effects of TEB on the parotid salivary gland. Its findings show morphological, molecular, and structural alterations in line with a progressive toxic picture following the coherent biological mechanisms. In addition to finding the parotid gland as a possible new target organ, this study proposes a model in which events such as oxidative stress, inflammation, apoptosis, and regenerative failures act interconnectedly, leading to loss of tissue integrity and function.

Oxidative stress represents the starting point of this pathological cascade, a classic and widely described early event for TEB‐induced toxicity. The significant increase in the immunostaining of 8‐OHdG, a marker of oxidative DNA damage, offers the molecular stimulus that suggests that exposure to the fungicide is related to the initial phase of cellular damage. This finding corroborates the literature, which has shown the genotoxic potential of TEB in multiple models. For example, TEB has induced lipid accumulation and oxidative stress in human HepG2 by increasing reactive oxygen species [10]. In a model with zebrafish, exposure to TEB also resulted in hepatotoxicity directly related to oxidative stress [19]. The same pattern occurs in mammals as studies on rats have shown that exposure to this fungicide causes oxidative stress, DNA damage, and apoptosis in the kidneys [20]. and reactive oxygen species‐mediated cardiotoxicity in the heart [20]. Moreover, intercellular erythrocytes show increased vascular permeability and possible endothelial damage, a finding compatible with the role of reactive oxygen species in modulating the capillary junction, as per studies on vascular permeability induced by oxidative stress [21].

This action that initiates DNA damage rather than acting in isolation triggers an inflammatory response, evinced in this study by the progressive increase in COX‐2 expression and the inflammatory infiltrate in the histopathological analysis. The literature strongly supports this correlation, as oxidative stress upregulates COX‐2 [22]. The expression of COX‐2 (a validated marker of inflammation in glandular tissues) markedly increases in salivary glands under xenobiotics, such as methotrexate [23]. Despite the scarce direct evidence in TEB‐exposed salivary glands, mechanistic support stems from studies showing that azole‐class fungicides can directly induce COX‐2 and TNF‐α expression in Sertoli cells. While this evidence is from an in vitro model, Sertoli cells have a glandular origin, suggesting a plausible parallel mechanism [24]. Moreover, TEB worsens inflammatory processes, such as autoimmune myocarditis in rats [25].

The progression of such cellular stress and inflammation culminates in irreversible damage: programmed cell death. In this study, the significant and statistically significant increase in the immunoexpression of caspase‐3 (one of the main executors of the apoptotic pathway) confirms the activation of this process in all treated groups. Othmène et al. [20] have shown that TEB increases the ratio between the pro‐apoptotic protein BAX and the anti‐apoptotic BCL‐2, leading to mitochondrial dysfunction and the sequential activation of caspase‐9 and finally caspase‐3. Interestingly, Lee et al. [26] studied mammary gland cells, showing the activation of caspase‐8, indicating that TEB can trigger the intrinsic and extrinsic pathway of apoptosis, generating a multifaceted pro‐death signal. Thus, the activation of this apoptotic pathway confirms the severe cell damage in the parotid gland due to the fungicide.

In the face of continuous aggression and cell loss, the parotid tissue showed signs of a complex adaptive response. We observed a significant elevation in the expression of CK7 (a structural protein of the glandular epithelium) at the highest doses. This constitutes a novel finding in TEB toxicity and suggests a phenotypic remodeling process in which surviving cells alter their characteristics to try to repair the damage or resist continued chemical aggression [10].

Notably, despite the intense apoptotic process, the expression of the proliferation marker Ki‐67 showed no significant differences. This result is particularly revealing when contrasted with in vitro evidence, which evinces an antiproliferative effect of TEB, decreasing viability and proliferation in mammary gland epithelial cells [26]. This apparent discrepancy points to a more complex in vivo mechanism, possibly a failed compensatory response. In this context, the high rate of apoptosis signals the need for cell replacement, stimulating an attempt at proliferation, a response reported in previous toxicity models [27]. However, the direct antiproliferative effect of the fungicide counteracts this regenerative response. This results in a seemingly unchanged level of Ki‐67 that likely masks the tissue's inability to mount an effective regenerative response. Chen et al. [28] corroborate this finding, reporting no change in the number of Leydig cells in adolescent rats exposed to TEB, indicating a similar lack of proliferative effect in that testicular cell type.

This failure in regeneration, added to the high rate of apoptosis, explains the significant and progressive reduction in cell density in the histomorphometric analysis, which begins at the lowest administered dose. Such tissue loss results from an imbalance between high cell death and insufficient proliferation. The ability of TEB to induce structural changes and compromise tissue integrity is an outcome consistent with its toxicity. Therefore, the cascade of molecular events, starting with oxidative stress and culminating in a failed regeneration, causes structural damage and loss of glandular tissue homeostasis. This pattern of damage does not seem to be an isolated event in TEB toxicology as Santos‐Miranda et al. [12] have shown similar patterns of structural and functional changes in murine heart tissue exposed to the same fungicide.

This process of tissue loss preceded a significant change in the extracellular matrix, as per the Picrosirius Red analysis. A dose‐dependent collagen accumulation showed a thickening of the perivascular and periductal regions, indicating fibrosis. This finding agrees with studies that showed the vulnerability of the parotid gland to systemic stressors. For example, Souza‐Monteiro et al. [29] have reported that chronic stress in rats also increases collagen deposition in this organ, confirming fibrosis as a common response pathway to damage.

Notably, the highest doses showed a predominance of thicker type I collagen fibers (orange‐red birefringence) associated with mature scars over thinner type III (green) related to flexible tissue. This pattern of collagen maturation toward type I strongly indicate the severity of the damage, a phenomenon also observed in human salivary pathologies. Using the same technique, Juengsomjit et al. [30] have shown that malignant tumors significantly increase type I collagen when compared with benign or chronically inflamed tissues. This transition to a rigid, fibrotic tissue represents the structural consequence of regenerative failure, in which non‐functional scar tissue replaces the lost functional glandular parenchyma.

In addition to the loss of structural tissue, the dose‐dependent response of PAS‐positive material in the parotid glands reflects a profound metabolic stress due to the high energy demand of the tissue to combat cell damage. The depletion of glycogen stores constitutes a classic toxicological response, a principle studies such as that of Mehana et al. [31] have validated as they show an analogous phenomenon of hepatic depletion in response to lead acetate, establishing that glycogen loss configures an expected marker of damage. In this study, this metabolic alteration is directly linked to initial oxidative stress. The literature strengthens this mechanistic connection since studies such as that of Toni et al. [32] have specifically associated TEB exposure with both the induction of oxidative stress and direct disturbances in the carbohydrate metabolism of carp. Therefore, the observed glycogen loss rather than an isolated event configures the energetic consequence of tissue effort to survive chemical aggression.

By finding the parotid salivary gland as a new and sensitive target organ, our findings fill an important gap in the literature and suggest that current safety parameters may be insufficient to protect against the full spectrum of its adverse effects. This reinforces the need to reassess safety limits, a concern reflected by agencies such as Health Canada, who reduced TEB application rates due to unacceptable risks of exposure in 2024 [7]. Its risk to human health is worrisome given its extensive use in several crops and its frequent detection in food [6], water, and even in the urine of exposed farmer populations, which confirms its systemic absorption in humans [8].

## 5 Concluding Remarks

This study provides overwhelming evidence that the fungicide TEB induces progressive toxicity in the parotid salivary gland, evincing it as a new and sensitive target organ. Results show an interconnected sequence of pathological events that begins with the induction of oxidative stress, progresses to an inflammatory response, culminates in cell death by apoptosis, and results in a regenerative failure that leads to structural tissue degradation, fibrosis, and decreased cellular activity. Therefore, this research suggests that current safety parameters may be insufficient to protect against the full spectrum of adverse effects from TEB, corroborating the need to reassess exposure limits, a concern already reflected by international regulatory agencies.

## Author Contributions

Study design: Gabriel Carvalhal de Aguiar, Lorrany da Silva Avanci, Daniel Vitor de Souza, and Lucas Vilas‐Bôas Correia. Data search: Gabriel Carvalhal de Aguiar, Lorrany da Silva Avanci, and Daniel Vitor de Souza. Data analysis: Gabriel Carvalhal de Aguiar, Lorrany da Silva Avanci, Daniel Vitor de Souza, and Daniel Araki Ribeiro. Data interpretation: Gabriel Carvalhal de Aguiar, Regina Cláudia Barbosa da Silva, Andrea Cristina de Moraes Malinverni, Fernando Augusto Cintra Magalhães and Daniel Araki Ribeiro. Writing the paper: all authors.

## Ethics Statement

This study was approved by Animal Ethics Committee under protocol no. 8359050722.

## Conflicts of Interest

The authors declare no conflicts of interest.

## Data Availability

Data sharing are available to this article upon request.
